# Concepts, Diagnosis and the History of Medicine: Historicising Ian Hacking and Munchausen Syndrome

**DOI:** 10.1093/shm/hkw083

**Published:** 2016-10-06

**Authors:** Chris Millard

**Keywords:** Retrospective diagnosis, Ian Hacking, Munchausen syndrome, anthropology, sociology, Erving Goffman, psychopathy, illness behaviour

## Abstract

Concepts used by historians are as historical as the diagnoses or categories that are studied. The example of Munchausen syndrome (deceptive presentation of illness in order to adopt the ‘sick role’) is used to explore this. Like most psychiatric diagnoses, Munchausen syndrome is not thought applicable across time by social historians of medicine. It is historically specific, drawing upon twentieth-century anthropology and sociology to explain motivation through desire for the ‘sick role’. Ian Hacking’s concepts of ‘making up people’ and ‘looping effects’ are regularly utilised outside of the context in which they are formed. However, this context is precisely the same anthropological and sociological insight used to explain Munchausen syndrome. It remains correct to resist the projection of Munchausen syndrome into the past. However, it seems inconsistent to use Hacking’s concepts to describe identity formation before the twentieth century as they are given meaning by an identical context.

## Introduction

Munchausen syndrome describes ‘the patient who chronically fabricates or induces illness with the sole intention of assuming the patient role.’^1^ Another recent report defines it as: ‘characterized by the intentional production or feigning of physical or psychological signs or symptoms, with a psychological need to assume the sick role.’^2^ The idea of a ‘sick’ or ‘patient’ role is central. In clinical circles it is more common today to refer to this condition as Fabricated or Induced Illness (FII); patients who might have been diagnosed with Munchausen syndrome are now positioned at the extreme end of an FII spectrum.^3^ Despite this, ‘Munchausen syndrome’ is used here because it is the dominant name for the condition in the middle of the twentieth century in the Anglophone states of the UK, Eire, the USA, Canada and Australia.

Munchausen syndrome is named by London physician Richard Asher in the *Lancet* 1951. He describes people arriving at hospitals reporting acute abdominal pain, mysterious bleeding or fits and headaches. After much investigation, these ailments are discovered to be consciously and deliberately deceptive. While the ‘sick role’ is a well-established part of the motivation today, this is not the case in 1951. Asher is almost wholly unable to account for the motivations behind these chronic deceptive presentations of illness. Instead he writes of ‘the apparent senselessness of it’ and roots it in an unspecific ‘strange twist of personality’ and ‘psychological kink’.^4^

Munchausen syndrome can be analysed as part of historically specific environments: matched up with institutional practices, medical ideas or cultural currents. In this case, the appearance of the syndrome is bound up with longstanding concerns around malingering and public funds, which are amplified by anxieties over the freshly-inaugurated National Health Service (NHS).^5^ However, this is not a parochial British story. The diagnosis travels far and wide (appropriately enough), being used in Eire in the early 1950s, the USA and Canada by the mid-1950s, and Australia and India by the 1960s. Historians of medicine have been documenting historical links between diagnoses, diseases and material and intellectual contexts for some decades.^6^ Adrian Wilson’s 15-year-old survey of these approaches remains one of the clearest statements of this historical method.^7^

Some of the most influential analytical tools used when analysing the history of diagnoses are those refined and developed by Ian Hacking from the early 1980s onwards.^8^ Hacking draws upon Michel Foucault to show how categories relating to selfhood and identity are intimately connected to specific historical circumstances. He begins his career as a philosopher of science, but migrates towards histories of psychiatric and medical classification, investigating multiple personality disorder, fugue states, and most recently, autism. It is this cluster of work around diagnosis that interests me: how diagnosis intervenes upon a person’s sense of self, and how that self might change. These insights are regularly deployed by scholars in the history of medicine.^9^ The two most relevant parts of Hacking’s conceptual armoury are ‘making up people’ and ‘looping effects’.

‘Making up people’ describes how people come to inhabit the identities that exist at various points in history, or in different enviroments. Hacking opens with the Foucualdian argument that ‘homosexuals’ did not exist before the nineteenth-century. The action of ‘sodomy’ is available before then, but the *type of person*, a ‘homosexual’ is not. Thus, the argument goes, people can only ‘make themselves up’ according to the possibilities for identity available in their particular context.^10^ ‘Looping effects’ is the name for what happens when people are labelled as a kind of person, or with a certain illness. It describes the ways in which people might react (or not) to that label. They can change their behaviour, they can be indifferent to the label, they can resist it, or they can adapt to fit it, and even to exaggerate it. Thus, Hacking describes how the revival of the diagnosis of multiple personality disorder (MPD) in North America in the 1980s leads, over time, to an increase in the average number of alternate personalities (‘alters’) thought to exist within individuals with MPD. The behaviour of those labelled ‘loops back’ onto the disorder once it is diagnosed, and then changes (exaggerates) the disorder itself.^11^ Hacking calls this ‘dynamic nominalism’, which describes the naming and the changes.

However, Munchausen patients characteristically fail to change in this way. Anthony Fry and Tania Gergel’s recent article on factitious disorder (FD) shows this rather clearly: ‘FD patients exhibit an inability to realize and accept the discovery and evidence of their condition. … This strongly suggests both a lack of insight and control over their condition’.^12^ A minority of patients actually do accept that they are faking, or simply stop their deceptions.^13^ The fact that some small number of patients might, against expectation, accept that they are simulating does not change that fact that Munchausen syndrome in general is characterized by an absence of acceptance. Whilst such repetitive and intractable patients might conform quite precisely to the doctor’s view of a Munchausen case (re-presenting over and over at hospitals), they are not ‘making themselves up’ as Munchausen syndrome patients, they are attempting to inhabit a different diagnosis altogether. Hacking allows for people to resist, to accept and to even be indifferent to diagnosis (in cases of autism, for example). All of these fall under the processes of ‘looping’. However, what is happening in Munchausen syndrome is a subversion of diagnosis and of identity formation *in itself*. Whilst this *is* a reaction to diagnosis, and may well qualify as ‘looping’, the focus here is that Munchausen syndrome subverts an important part of this process of identity formation (the self-conscious negotiation), as well as being another variety of it.

Why is it important that Munchausen syndrome is a subversion of diagnosis, rather than resistance or acceptance or indifference? The answer is disarmingly simple, but it has complicated implications. Munchausen syndrome becomes understood as disordered identity, as a pathological adoption of a social role (the ‘sick role’). This means that it identifies precisely a *disorder* of part of those role-adopting processes that ‘looping’ and ‘making up people’ seek to *describe*. The part of ‘making oneself up’ that describes self-conscious identity formation is understood as the site of the pathology. This unites Munchasuen syndrome and ‘making up people’, showing them to be equally situated and historical. Munchausen syndrome emerges at a time when connections between medicine, sociology and anthropology are growing. Awareness of a person’s ‘social setting’ is high and sociological frames of reference help doctors to categorise and understand these baffling patients. Initially this leads to a focus on their ‘anti-social behaviour’. As we approach the 1970s, ideas of sociological roles in medicine start to be applied more explicitly to what is then called ‘abnormal illness behaviour’.

The implications of this are broad. It is not that Munchausen syndrome causes Hacking’s insights to ‘fail’. The point emphasized here is that these connections with twentieth-century sociology and anthropology are what make it illegitimate to project Munchausen syndrome back into the past. Without those historically specific intellectual insights around ‘roles’, Munchausen syndrome cannot exist in any meaningful way. However, ‘making up people’ and Munchausen syndrome are based upon exactly the same sociological insights. It is not simply the case that Munchausen syndrome is bounded by a context that is *analogous* to ‘making up people’: Munchausen syndrome is bounded and situated by *precisely the same context*. Thus the example of Munchausen syndrome makes it most obvious that Hacking’s concepts are *also* historical objects, and should not be thought useful without limit and outside history (constraints readily accepted for psychiatric diagnoses such as Munchausen syndrome). They are both part of a particular way of seeing the world that is influential at a certain time. Hacking is explicitly influenced by sociologists and anthropologists—particularly Erving Goffman—and it is this cross-fertilisation that undergirds his analysis of the malleable, performed nature of identity. There are other, similar innovations in psychology, philosophy and linguistics. Rather than seeking to diminish Hacking’s signal contributions, this article aims to come to terms with the fact that the ideas, tools and concepts used *in* history are *as historical* as the ideas and categories they are used to study. Taking this further, it appears anachronistic to apply ‘looping effects’ to medieval saints or ancient Greeks. Why should concepts forged through interactions between sociology, philosophy and medicine in the twentieth century be apt to describe identity formation throughout history and across cultures?

## Sociology and Medicine—Broad Interactions

Munchausen syndrome is initially baffling because the doctors cannot see what motivates these patients to undergo countless painful and dangerous operations. Asher argues that ‘Unlike the malingerer, who may gain a definite end, these patients often seem to gain nothing except the discomfiture of unnecessary investigations or operations.’^14^ This absence of gain slowly recedes as Munchausen becomes haltingly understood through an awareness of the satisfactions of the social role of the patient, and thus through the increasing connections between sociology and medicine. This is part of a much broader shift. Erstwhile General Practitioner and influential sociologist of medicine David Armstrong speaks of a twentieth-century ‘new gaze [which] identified disease in the spaces between people, in the interstices of relationships, in the social body itself’. He draws attention to the social focus of medicine in this period, adding that ‘[i]n psychiatry, sociology has provided a rich and diverse contribution to the extension of the medical gaze’.^15^ Social settings, social environments and social relationships all become influential in medical practice in new and important ways. Armstrong traces this shift back to the early twentieth century, but it is also clear that the Second World War has an accelerating effect. As Colin Jones puts it: ‘the impact of Hitlerism deterred psychiatrists from locating mental health problems solely within the individual and pushed them towards more societal explanations’.^16^

There are significant developments in academic sociology. American sociologist C. Wright Mills’s classic *White Collar* (1951) claims that ‘[w]e need to characterize American society of the mid-twentieth century in more psychological terms, for now the problems that concern us most border on the psychiatric’.^17^ Sociology and psychiatry are mutually reinforcing here. A key staging-post in medicine and sociology’s interaction is sociologist Talcott Parsons’ concept of the sick role, with its attendant benefits and costs. Parsons’ analysis is published in 1951—incidentally the same year as Asher’s article on Munchausen. As historian John C. Burnham points out, the ‘sick role’ is not wholly Parsons’ idea—he builds upon (but does not acknowledge) the work of a Harvard graduate student, David M. Schneider. Schneider studies behaviour around illnesses in army units during the 1940s, showing the roots of Munchausen syndrome in anthropology and malingering.^18^ It is also clear that, as Burnham affirms, the ‘primordial source for the idea of the sick role was cultural anthropology’.^19^ Thus twentieth-century sociology and anthropology—from Mills and Parsons, and back through Herbert Blumer, Clifford Geertz, Margaret Mead, Gregory Bateson and others—is centrally implicated in Munchausen syndrome.

Parsons identifies four aspects to the ‘sick role’, and casts them in terms of exemptions on the one hand, and obligations on the other. Anyone who has the sick role is exempt from normal social obligations, exempt from responsibility for the sickness, obliged to try and get out of the sick state as quickly as possible, and required to seek appropriate help to do so. Munchausen syndrome patients come to be seen as desiring the exemptions but not fulfilling the obligations. However, we must also be aware of Armstrong’s point that ‘within Parsons’ analytic framework there was little space for independent action by the patient’. The Parsonian vision of the patient, according to Armstrong, is that of ‘docile figures with no responsibility for their predicament and minimal involvement in their own care’.^20^ This is perhaps why the ‘patient role’ does not initially seem to fit Munchausen, as it involves rather more agency on the part of the supposed patient. Another important conduit for crossovers between sociology, anthropology and medicine is Erving Goffman’s work. His influential works include *The Presentation of Self in Everyday Life* (1957), *Asylums* (1961) and *Stigma* (1963).^21^ As we shall see, Goffman’s influence on Hacking emerges in a significant way in the 1980s, in the same way as it becomes useful to understandings of Munchasuen patients in the 1970s.

However, what is most interesting here is how Goffman’s earliest work analyses ‘confidence tricksters’, or ‘con-artists’. Historian of psychology Michael Pettit astutely observes that this feeds into Goffman’s view of ‘social intercourse as an inherently deceptive dramatic performance’.^22^ At the centre of Goffman’s sociology is a dissimulating human nature. Tightly intertwined here are post-war sociology, medicine and a concept of human nature that corresponds to Munchausen syndrome remarkably well. A recent article reviewing factitious disorders in the *Lancet* echoes this sentiment, showing this perspective—the normalisation of deception—to be influential still. This article claims that ‘although factitious disorders and malingering are both clinically significant, deception is a pervasive, normal, and ubiquitous social behaviour of human nature.’^23^

Munchausen syndrome travels from the UK to North America through correspondence in medical journals, an article in *Time* magazine in 1951, as well as an editorial in the *Canadian Medical Association Journal* in the late 1950s. In a similar way, sociology practised and popularised in North America by Talcott Parsons, C. Wright Mills and Erving Goffman also travels, and influences academics and physicians in Britain. This does not happen only through vague Anglophone academic osmosis. Goffman’s doctoral fieldwork in anthropology is carried out in the mid-1950s on the Shetland Islands, off the coast of Scotland, under the auspices of the University of Edinburgh. In addition, pioneering medical sociologist David Mechanic completes a year’s fellowship in the Social Psychiatry research unit at the influential Institute of Psychiatry and Maudsley Hospital in South London in 1965–66.

In 1960, Mechanic and Edmund Volkart put forward the concept of ‘illness behaviour’, by which they mean ‘the ways in which given symptoms may be differentially perceived, evaluated, and acted (or not acted) upon by different kinds of persons’.^24^ Here again, the behavioural sciences and their interaction with medicine is key. It is not just illness, but the subjective experience of illness, and the attitudes of the people so diagnosed that is the focus here. This sociological and anthropological influence is explicitly built upon by Issy Pilowsky in 1969, who adapts these insights to analyse what he calls ‘abnormal illness behaviour’—which includes Munchausen syndrome.^25^ Pilowsky brings together Parsons, Mechanic and psychiatry (specifically discussions of hysteria), joining up the dots between sociological roles, ideas of behaviour and psychopathology (or at the very least, psychological abnormality).

Eminent psychiatrist Martin Roth delivers an entertaining Presidential Address to the Medical Society of the University of Durham in 1962 on the topic of the ‘Desire to be Ill’. In it, he argues that ‘explanations for the deliberate simulation of illness or disability … would require much more knowledge than we possess’. However, he does see a number of professionals being able to contribute: ‘the field is of sufficient interest to be worthy of research by the psychiatrist, the physician and the social scientist’.^26^ This is an early example of a non-clinical worker (and social scientist in particular) being put forward to explain the baffling motivations, showing the potential for overlap between social science and medicine.

David Vail, a doctor at the Department of Public Welfare in Minnesota, USA, describes two cases in his 1962 article ‘Munchausen returns’. He notes that ‘A previously unexplored dimension in these cases is the sociological’ and that ‘just as there are well-established modes, according to which class distinction and behavior operate among hospital staffs, there are definite rules for patients also.’ Vail also mentions that one of these men ‘has adapted himself to hospitals’.^27^ It must be said that Vail’s use of the term ‘sociological’ here seems a little idiosyncratic, and he does not talk of ‘roles’, but does use the sociological staples of culture, adaptation and adjustment. It is clear that the analytical frame is zooming out, taking in the environment and the psychopathology of these patients. More generally, the idea of role play seems to gather pace during the 1960s, as a Munchausen report from Chandigarh, India draws upon (and footnotes) Asher to argue that one motive is ‘[a] pathological satisfaction derived from playing the role of a patient’.^28^

Patient presentations and roles are also understood in a commonsense idiom. In 1963, Robert Kemp writes in the *Lancet* of the ‘familiar face syndrome’ or the ‘thick-file case’, and cautions that one ‘should bear in mind what is often pointed out by the novelist, that ill health can be a gainful (even if disastrous) policy in itself’.^29^ In 1964, one Surrey doctor writes to the *British Medical Journal* describing ‘those people whose main role in life has in fact become that of a patient. Some of these people seem to have adopted this role almost as a conscious choice in distinction to following some gainful occupation or profession.’^30^ Both examples acknowledge the positives involved in patienthood. ‘Sick roles’ *become* integral to Munchausen patients through an appropriation of sociological knowledge into psychology and psychiatry. Sometimes this is explicitly borrowed, as in the cases of Vail and Pilowsky, and sometimes it is not. As we have seen above, it is now common to invoke these role aspirations as central.

## Psychopathy and Anti-social Behaviour

Cross-fertilisation with sociology, anthropology and social science is not the *only* way that Munchausen syndrome and deceptive health behaviours are understood. When Munchausen syndrome first emerges, it is chiefly labelled as a variant of psychopathy. Eric Frankel from Wanstead Hosptial argues in 1951 that ‘[t]hese patients are invariably severe psychopaths, and their psychopathic personality requires treatment’.^31^ Two doctors in Hammersmith in 1958 are relieved that ‘these patients have at last been recognized as a special type of psychopathic personality’.^32^ Today the term ‘psychopath’ conjures up the caricatured, fictional extremes of Hannibal Lecter and Patrick Bateman—men who are charismatic, violent, ruthless and successful. In contrast, during the 1950s and 1960s psychopathy covers a much wider range of supposed conditions, linked by their apparent antisociality, including homosexuality, delinquency, alcoholism and chronic unemployment.^33^

The diagnosis is rather nebulous. As Maxwell Jones states in expert evidence to the Percy Commission (the recommendations of which form the basis for the Mental Health Act 1959): ‘It is probably impossible to find a satisfactory definition for psychopathic states at the present state of our knowledge.’^34^ The British Medical Association’s memorandum to the same Commission says that they ‘considered the question of formulating a definition of “psychopathic offender” but reached the conclusion that it would be unwise to attempt this as such a definition might prove difficult to operate in a court of law’.^35^ More succinctly, Barbara Wooton sums up the available knowledge in 1959, concluding that ‘psychopaths are extremely selfish persons and no one knows what makes them so’.^36^

Despite these definitional difficulties, psychopathy is inextricably bound up with ideas of the ‘social setting’. Martyn Pickersgill argues that during the 1960s in Britain ‘[t]here was agreement at least that psychopathy was socially, as well as clinically, problematic’.^37^ In fact, anti-sociality is right at the core of descriptions of psychopathy in this period. Jones’s memorandum to the Commissioners describes a ‘concept of social defectiveness as … more realistic than any attempt at a diagnostic classification’.^38^ The British Medical Association opens its section on psychopathic states by talking of ‘mental abnormality’ which renders people ‘delinquent or otherwise anti-social’. This is repeated by the Royal Medico-Psychological Association—the forerunner of the Royal College of Psychiatrists.^39^ The connections between psychopathy (and thus Munchausen syndrome) and social science go deeper. When describing a treatment programme specifically for psychopaths run at Belmont hospital, Jones reports using ‘many concepts borrowed from the social science field’. Moreover, when questioned, he reveals the extent of ‘social science’ involvement: ‘We have largely dispensed with orthodox nursing help … and orthodox psychiatric treatment … [A]part from a nucleus of trained nursing staff, we now use social science personnel. We have eleven people most of whom have a social science training rather than a training in the field of medicine’.^40^ The ‘social’ over the ‘medical’ is explicit here.

A 1959 editorial in the magazine *Medical World* puts this in critical terms: ‘the criteria of psychopathy are social not medical. Doctors should not be asked to act as the social conscience of society’. Psychopathy is seen—explicitly by some—to be an extension of the social role of medicine: society’s social conscience.^41^ Finally, an obscure article in a sociological journal that predates Asher’s Munchausen article *and* Parsons’ *Social System* gives an insight into attempts to understand psychopathy wholly in terms of social roles. ‘A Sociological Theory of Psychopathy’ (1948) by Harrison G. Gough builds on the anthropology of George Herbert Mead and others to argue that ‘the psychopathic personality is pathologically deficient in role-playing abilities’.^42^ This shows the links between an appreciation of the social setting and newly prominent forms of mental pathology. These are used to shed light upon those labelled as having Munchausen syndrome. This idea of psychopathy being ‘social’ in this specific historical period can be usefully contrasted with one of the ideas ‘gaining credence today’ according to Pickersgill, that ‘psychopathy is a disorder of empathy and hence related to the amygdala.’^43^ Psychopathy isn’t *necessarily* social, but it most certainly is at this historical juncture, and this is key to understandings of Munchausen syndrome in the 1950s.

Munchausen syndrome is not *solely* understood through a socially-focused concept of psychopathy, or only through anthropological and sociological concepts of roles. However, these are influential ways of doing so from the 1950s onwards. There are a number of other concepts such as ‘secondary gain’ and ‘imposture’, which are popular within psychoanalysis, as well as understanding it in relation to models of anorexia nervosa and addiction.^44^ However, it is striking is that even before Munchausen syndrome is understood in an explicitly sociological (role-centred) manner, it is still understood as an exceptionally social—in fact ‘anti-social’—phenomenon.

## Hacking and Goffman—Looping and Sociology

Having shown the diverse links between Munchausen, psychopathy, sociology and the social setting, it remains for me to link up the other half of the argument—the concepts of Ian Hacking. The aim is to show that both Munchausen syndrome and ‘making up people’ are built on the same sociological/anthropological foundations, part of the same historically situated problem. Thus if Munchausen syndrome is not to be projected backwards and used to understand behaviours before the 1950s, it is inconsistent to perform the same projections of ‘making up people’ before the emergence of the sociology and anthropology upon which (as we shall see) it is based. This is *not* to claim that Hacking’s concepts fail, but instead that they are historical, in precisely the same way as Munchausen syndrome.

First, we must just glance at two of the most recent and clear statements on looping. In Hacking’s review of the fifth edition of the *Diagnostic and Statistical Manual of Mental Disorders* in 2013 he notes that diagnosis has

[S]ubtle effects on how patients think of themselves, how they feel and how they behave. Especially since nowadays … patients tend to look it up online. There they obtain a sort of stereotype of how they ought to be feeling and behaving.^45^

In an interview after winning the Holberg Prize he states that ‘I do think there is a widespread phenomenon I called “looping”. Classifying people has an effect on how they conceive of themselves, they internalize how they are classified’. The example cited is that of MPD, where patients ‘adapted their behaviour to fit the diagnosis’ but also exaggerated it. As noted, Hacking demonstrates the inflation in the average number of personalities that patients have, showing how ‘the behaviour of the patients looped back on the description of the disorder’.^46^ In a general sense, he argues that ‘[c]lassifying changes people, but the changed people cause classifications themselves to be redrawn’.^47^ These passages all involve identification with a diagnosis, and an internalisation of it. To the extent the diagnosis changes, it becomes more extreme—the opposite of resistance.

Hacking’s essay ‘The Looping Effects of Human Kinds’ also mentions a stereotypical response to being labelled as a ‘bad kind’. Here looping involves a move away from the negative connotations, with Hacking talking about how people might ‘do things a little differently from now on. Not just to escape opprobrium … but because I do not want to be that kind of person’.^48^ This *is* resistance in a way, but based around an awareness that a category is ‘bad’ and acceptance that it is not good to be classed as one of those. There is still an identification with the value system, an internalisation of the prohibition. Finally, Hacking mentions what he calls ‘inaccessible kinds’—such as infants or children diagnosed with autism—who ‘cannot take in how they are classified’. In these cases the looping effect ‘works on the kind and its auxiliaries—family and remedial workers’.^49^

People might resist, they might accept, they might exaggerate or they might be indifferent. In all cases, looping still occurs. Hacking describes many varieties of ways to react, but Munchausen syndrome is different, as it explicitly pathologises part of those processes: the attempted assumption of one identity (acute physical sickness) and self-conscious resistance of another (the dissimulating, psychopathic Munchausen patient). Looping is built upon the same (historical) insights as those that structure Munchausen syndrome, it is in fact a pathological manifestation of them. Thus Hacking’s concepts and Munchausen syndrome can be thought of as conceptually bolted together, as the normal/pathological sides of the same anthropological coin. However, before we get to the significance of this subversion, we should assess Hacking’s explicit links to earlier sociology, especially that of Goffman and Parsons. Parsons’ functionalist sociology of 1951 is worlds away from the sophisticated dynamic nominalism of Hacking from the early 1980s onwards, but the links between sociology and anthropology and Hacking’s work are strong. As the ‘sick role’ becomes increasingly central to Munchausen syndrome in the 1970s and 1980s, that same sociological awareness of the flexibility and performativity of human social interaction informs ideas of ‘making up people’ and ‘looping effects’. It is right at this time (the early 1980s) that Hacking begins a transition from his work in the history of science to working on subjectivity. The paper ‘Making up People’ is given in 1983, but the previous year he publishes a paper on ‘Biopower and the Avalanche of Printed Numbers’.^50^ This paper might tentatively be seen as marking a transition between Hacking’s histories of science and his histories of subjectivity. His book *The Emergence of Probability* (1975) is firmly in the history of science mould, and deals with statistical reasoning.^51^ He then extends his statistical interests to those labelling humans, talking about suicide statistics. Here he clearly signals the influence of Michel Foucault—perhaps the most famous twentieth-century scholar of subjectivity—by using the word ‘biopower’. In fact he began that article by mentioning Foucault’s 1966 classic *Les Mots et Les Choses*, rendered into English as *The Order of Things.*

However, Foucault is not the influence at issue here, but various sociologists and anthropologists, chief amongst them Erving Goffman. Hacking not only acknowledges but celebrates his debt to Goffman. A review of two of Hacking’s books in 2001 notes that:

Goffman developed an ecological orientation and deployed a conceptual vocabulary (involving ‘looping effects’) that resembles Hacking’s substantive account of transient mental illnesses. Sociologists who read Hacking may appreciate his substantive research but wonder if he is not reinventing the wheel. Or, rather, they may wonder if he is not reinventing *the loop*.^52^

Lynch admits that if taken too literally, this ‘would be unfair’ but that ‘it is worth exploring the parallels’.^53^ A few years later, Hacking responds generously: ‘How right he was! I have a slightly different view of the parallels than he does; I think the parallels are like straight lines that never meet.’^54^ Thus ‘looping’ is tightly intertwined with a particular period in sociology.

## Munchausen Syndrome and Looping: Anthropology and ‘Malleable Humanity’

Patients with Munchausen syndrome are increasingly understood through the social setting, psychopathy and social roles. We have also seen how specific, influential sociologists are central to Hacking’s work. It has also been shown how Munchausen syndrome patients complicate ideas of looping because the patients’ motivations are understood as a pathological form of exactly the same sociological processes that undergird Hacking’s concepts. Both are based on a universal vision of human nature that is plastic, malleable and inscribable—this idea emerges directly from early twentieth-century anthropology.

It is important to note how Hacking explicitly disavows a certain kind of universalism:

Philosophy is heroic (in my version of events) when it tries to paint a picture of the whole of human nature—and of the place of human beings in nature. … I am the very opposite of heroic, not cowardly but proudly particularist. I think there is no fixed whole of human nature to discuss.^55^

This point about particularism, along with his statement that there is ‘no reason to suppose that we shall ever tell two identical stories of two different instances of making up people’ is well taken.^56^ However, Hacking does not say anywhere—so far as I am aware—that people will not loop, even if that looping is of a non-self-conscious (inaccessible) type. Indeed, it might even be said that the subversion of looping by attempting to assume the sick role is a kind of looping after all, because the diagnosis of Munchausen syndrome affects those around the patient, whose behaviour then changes. In this way, there is no fixed human nature for Hacking precisely because everybody loops, in different contexts, at different times, with different interactions and varied stimuli. Or to put it another way: nobody is fixed because everybody loops.

Hacking’s concepts cover an enormous variety of practices, but in the end, everybody loops, anywhere, at any time. This in itself should ring alarm bells for historians, as it seems to be a trans-historical or extra-historical process. The very variety spawned by these concepts relies upon a universal plasticity, which props up a universally interacting, looping selfhood. This universally plastic selfhood (or perhaps ‘pre-self’) has its source in a specific kind of twentieth-century anthropology. Margaret Mead’s classic *Coming of Age in Samoa* (1928) celebrates the manifold differences in the process of adolescence (‘coming of age’) between North America and Samoa. The book is also explicitly addressed at the problem of juvenile delinquency in the United States, and how this might be solved—one of the later chapters is entitled ‘Our Educational Problems in Light of Samoan Contrasts’.^57^

The relevance of Mead’s work in Samoa to the problem of juvenile delinquency in the USA rests upon this sense of a universal plasticity to human nature. Mead’s mentor, Franz Boas, writes an appreciative foreword in which he claims ‘much of what we ascribe to human nature is no more than a reaction to the restraints put upon us by our civilization’.^58^ Mead herself writes that ‘neither race nor common humanity can be held responsible for many of the forms which even such basic emotions as love and fear and anger take under different social conditions’. But straight from this disavowal of common humanity, she deploys something universal, writing of ‘babies who have as yet no civilization to shape their malleable humanity’.^59^

Thus, Mead’s argument runs, if some of the features of Samoan society that supposedly render ‘coming of age’ so serene in the Pacific could be transferred to the USA, it might ameliorate the delinquency problem. Precisely because this anthropology must parse so much difference (and indeed it implicitly attacks the idea that problems like ‘juvenile delinquency’ are inevitable or universal), it relies upon this ‘malleable humanity’: an overarching sameness, a human nature that is fundamentally *inscribable*, fundamentally moulded by circumstance. Australian anthropologist Derek Freeman has spent a great deal of time and energy criticising Mead’s fieldwork in Samoa, and what he calls ‘Boasian culturalism’ in general. This latter he characterises as the ‘extreme’ and ‘preposterous’ conclusion that ‘the phenomena of adolescence are due not to physiology, but to “the social environment”’. Preposterous or not, these ideas have been very influential in certain circles. Freeman admits as much, quoting an historian of American anthropology who in 1973 characterised such ‘culturalism’ as ‘fundamental to all of American social science’.^60^

Thus sociology, social science, psychopathy and Munchausen syndrome all draw upon the same set of insights and conceptual frameworks as Ian Hacking, Erving Goffman and even Michel Foucualt. Rhodri Hayward notes that Foucault’s arguments (made in the 1960s) around moral treatment in nineteenth-century asylums are built upon the idea that the ‘patient’s psyche was a *tabula rasa* inscribed with the interests of lay asylum keepers, medical professionals, magistrates and their desperate families’.^61^ This ‘malleable humanity’ in Mead’s apt phrase is precisely the root of Hacking’s denial of a universal human nature. Ironically enough, this anti-universalism is based upon an idea of a universal plasticity.

Munchausen syndrome and ‘making up people’ and ‘looping effects’ are thus shown up as parts of the same intellectual context. Patients with Munchausen syndrome subvert part of what Hacking describes (in all its detail and variation). It is all built upon historically situated anthropological frames of reference.

It might be asked whether Munchausen syndrome, alone amongst diagnoses, can make obvious the situated nature of Hacking’s concepts. In one sense that answer must be ‘no’ because all diseases have specificity, and all diagnoses are situated and historical, from schizophrenia to syphilis, from pleurisy to post-traumatic stress disorder.^62^ What is important here is that Munchausen syndrome brings the situated nature of Hacking’s concepts into sharp relief because it is bounded and historically specific *in the same way* as Hacking’s concepts are.

Similarly, arguing that Munchausen syndrome is diagnosed through a process of ‘making up people’ should not be taken to imply that this syndrome is the only (or even the dominant) identity for these patients. Identity is malleable and context-specific in this reading: Munchausen syndrome forms around the self that interacts, deceives and negotiates in the emergency department. Asher notes ‘an intense desire to deceive everybody as much as possible. … They lie for the sake of lying … merely from a love of falsehood.’^63^ This suggests that the deception is not confined to hospital presentations that become labelled as Munchausen syndrome.

Built around notions of identity, through a flexible and specifically anthropological understanding of social roles, Munchausen syndrome brings the specificity of Hacking’s methodological insights into sharper focus than perhaps any other diagnosis or illness category. Munchausen syndrome shows most clearly that the historical baggage around a disease category is of the same quality as the baggage around Hacking’s concepts because *it is the same baggage*. Instead of being able to show that a disease category’s historical specificity is *analogous* to that of Hacking’s concept, Munchausen syndrome enables me to show that it is *precisely the same* specificity as that which is attached to Hacking’s concepts. Armed with this insight, we can analyse the historical nature of analytical concepts. John Savage and others have termed this the ‘social life of methods’, where methods are ‘a fascinating object of inquiry … the very stuff of social life.^64^ Diagnoses *and* the ways in which we analyse them are historically contingent. Hacking’s concepts and Munchausen syndrome are based on the same anthropological and sociological insights, standing upon the same intellectual ground. This particular way of seeing the world brings out the self-conscious, performative, aspects of human interaction.

## Twentieth-century Identity

It is a characteristically twentieth-century position to view human nature as self-conscious acting. This involves rejecting the idea of a truth or essence, coming to believe instead that the truth is malleable, and identity is dynamic (and therefore might loop, or become disordered). This radical scepticism around the possibility of essences can be seen in Jacques Derrida’s theory that words have no essential meaning; meanings are conveyed by the relations between words.^65^ Stuart Hall memorably appropriates this insight when talking about concepts of race:

*race works like a language* … things gain their meaning, not because of what they contain in their essence, but in the shifting relations of difference. … Their meaning, because it is relational, and not essential, can never be finally fixed, but is subject to the constant process of redefinition and appropriation.^66^

This rejection of essences, and its relation to Munchausen syndrome and Ian Hacking, can be seen perfectly, if unexpectedly, in a *Lancet* lead article from 1962. This article, in a prominent medical journal, draws out the startling implications of Jean-Paul Satre’s existential philosophy for malingering patients. Entitled ‘Compensation for Cupidity?’ the article approaches the question of financial compensation for psychological injuries. As noted, Munchausen syndrome has roots in concerns about malingering and fraudulent claim-staking. The article claims that ‘one of the causes of such traumatic neurosis is the expectation of compensation, and that the law is paradoxically compelling one party to compensate another for a consequence of his act which the law itself has created’.^67^ Therefore, the provision of compensation encourages and indeed *produces* the psychological injury. Rather than decrying this as greed or fraud, the author notes instead that this brings us to ‘the root of human responsibility’ and draws upon analysis of Jean Paul Sartre: ‘The essence of man … lies rather in the radical liberty of man’s existence by which he chooses himself and so makes himself what he is. … A man is his life, says Sartre.’^68^ People perform their selves, and their selves are coterminous with their actions. This view is by no means a typical one for a physician to hold, but its presence in a medical journal is intriguing.

Identity is here radically malleable, in a context where people are presenting illness or injury. This context, where identity is seen as non-essential and performed, resonates profoundly with Munchausen syndrome. Rhodri Hayward’s study of psychological approaches in twentieth-century British general practice ends with a similar disavowal of simplistic notions of truth. He argues that instead of ‘unrelenting scepticism’ towards ideas of truth we might focus on how, in the ideas of twentieth-century psychiatrists ‘the mere fact of belief is transformative … capable of creating new illnesses, new kinds of patients and a new vision of society. It made its own truth.’^69^ This idea of the truth being made and achieved forecloses the possibility of identity being stable and fixed. This position is famously expressed by Sartre in *Being and Nothingness*, first published in English in 1956. Sartre notes that a waiter at a Parisian café performs his role, almost exaggerating it, in order to be what he is:

the waiter in the cafe plays with his condition in order to realize it. This obligation is not different from that which is imposed on all tradesmen. Their condition is wholly one of ceremony. The public demands of them that they realize it as a ceremony; there is the dance of the grocer, of the tailor, of the auctioneer, by which they endeavour to persuade their clientele that they are nothing but a grocer, an auctioneer, a tailor. … There are indeed many precautions to imprison a man in what he is, as if we lived in perpetual fear that he might escape from it, that he might break away and suddenly elude his condition.^70^

This is a paradox. The idea of performing one’s self in order that one does not elude oneself is paradoxical because an essence cannot be an act, just as an act cannot be an essence:

[I]f I represent myself as him, I am not he … I can only play at being him … if I am one [a waiter], this can not be in the mode of being in-itself. I am a waiter in the mode of *being what I am not*.^71^

This paradox is analogous to the one at the centre of Munchausen syndrome, where a person is exposed as ill, precisely because they are pretending to be ill. More generally, according to Sartre, a person can only achieve their identity by play, self-conscious acting. During the mid-twentieth century, there is an increased awareness of acting, performing and imitating that explicitly rejects the idea of a deep, fixed self beneath it (as in Hacking’s disavowal of a singular human nature). The acting and deception becomes the self—but all in a very specific context. If Munchausen syndrome, anthropology and existentialism are context-specific, so must ‘looping’ be.

And here we complete the loop back to Hacking, who uses Sartre’s waiter in ‘Making up People’. He argues that

Thus the idea of making up people is enriched; it applies … to all of us. It is not just the making up of people of a kind that did not exist before: not only are the split [personality patient] and the waiter made up, but each of us is made up.^72^

‘Making up people’ fits within a web of twentieth-century ideas: medical discussions of compensation, psychology in general practice, post-colonial race theory, cultural anthropology, Derrida’s linguistics and Sartre’s existentialism. The concern of medicine with identity shows up most clearly in Munchausen syndrome, where roles, acting and identity collide. This is complicated by the fact that Munchausen syndrome becomes a psychiatric pathology. The patient ‘plays’ at being ill, and therefore achieves a (different) patienthood; the waiter ‘plays’ at being a waiter, and becomes one by virtue of what he is not.

The diagnosis of Munchausen syndrome is a paradoxical attempt to fix the identity of the patient. It is built upon the possibility that identities might be managed or controlled by patients, but also attempts to shut this down and police it. Far from Sartre’s radical freedom, most doctors diagnosing Munchausen syndrome seek to impose control, arguing for blacklists, photographs and issuing warnings in correspondence to aid other doctors in the swift exposure of such patients. Others seek to contain, treat and cure what they see as a dangerously self-destructive psychological disorder. This is the polar opposite of the *Lancet* article’s Sartrean acceptance. This refusal of flexibility attempts to confound the malleability in ‘making up people’: patients diagnosed with Munchausen syndrome are, by that act of diagnosis, denied the flexibility to assume the sick role. In fact, the attempt is pathologised. Again, this shows that Munchausen syndrome’s historicity is identical to Hacking’s.

Stephen Greenblatt’s famous idea of *Renaissance Self-Fashioning* (1980) emerged around the same time as Hacking’s ‘Making Up People’ (1983). However, according to the logic of my argument, it would be a mistake to equate practices of Renaissance people with the tangled twentieth-century threads of Sartre, Hacking and Munchausen syndrome.

In any case, Greenblatt explicitly acknowledges his debt to precisely the anthropology we have been discussing. He claims that his intention is to ‘practice a more cultural or anthropological criticism—if by “anthropological” here we think of interpretive studies of culture by [Clifford] Geertz, James Boon, Mary Douglas, Jean Duvignaud, Paul Rabinow, Victor Turner and others’.^73^ A malleable identity is read into the past through a very specific corpus of twentieth-century anthropology. This is not to demean Greenblatt’s scholarship, for he does acknowledge his own situatedness, accepting ‘the impossibility of … leaving behind one’s own situation … the questions I ask of my material and indeed the very nature of this material are shaped by the questions I ask of myself’.^74^ However he does not link this up to his twentieth-century anthropological tools. He also slips into rather totalising language at points. When discussing a particular passage of Spenser’s *The Faerie Queene* (1590, 1596) he argues that ‘The experience I have just described is, insofar as the work retains its power, common to us all, embedded in each of our histories personal histories, though a protective cultural amnesia may have led us to forget it until we reexperience it in art.’^75^ Thus Greenblatt uses psychological and anthropological concepts and approaches, created in the twentieth century, in order to understand texts from the sixteenth century. Further, upon this reading, he explicitly argues for a human universal: even if you don’t think you have experienced it, you are probably being protected by ‘cultural amnesia’.

All this might sound rather sniping, petty and unfair. I want to stress that I am not seeking to invalidate Greenblatt’s pioneering scholarship (and I am in no way pretending any expertise in Early Modern literature). In any case, it is highly significant that during a relatively short period—less than 15 years—between the late 1960s and early 1980s, all these examples emerged. We have the concepts of abnormal illness behaviour (1969), self-fashioning (1980) and making up people (1983) all drawing significantly upon anthropology and sociology. It is also during the 1980s that Hacking begins his shift from the history of science to the history of subjectivity. There are many more examples out there than cannot be presented in a short article. My point is to ask a more general question: given the clear specificity of this ‘malleable humanity’ in twentieth-century anthropology, which concepts are we happy to project back into the past, and at which ones do we baulk? It remains the case that historians of medicine generally give much shorter shrift to diagnoses of Hamlet as schizophrenic, than those who project anthropological (rather than medical) concepts back in time, into places where they did not exist. This point is taken up in conclusion.

## Conclusion

Thirty years on from ‘making up people’ we must historicise these ideas, and sketch their limits. Munchausen syndrome enables us to see how both ‘making up people’ and ‘Munchausen syndrome’ are based upon the same broad influential, *historical* idea: that identities and roles are malleable and manageable; they are deeply affected by self-consciousness; they are bounded and enabled by social context; they can be performed, manipulated, aspired to and achieved. Thus: they can become disordered. Why should concepts be ideal and ahistorical when we accept that diagnoses are not?

Although the work of Giles Deleuze and Felix Guattari is often infuriatingly opaque, there is something in their final collaboration *What is Philosophy?* that is useful when considering these limits. When interrogating the nature of concepts (in our case *either* concepts of Munchausen syndrome *or* of ‘making up people’ and ‘looping effects’) they argue that ‘[a]ll concepts are connected to problems without which they would have no meaning’. This emphasises the point that concepts are situated and ‘can only be assessed as a function of their problems and their plane’.^76^ This sense of limit, of a boundary beyond which concepts ‘have no meaning’ is what I want to emphasise. This question might also be approached through an appreciation of reflexivity, as formulated a decade ago by Roger Smith:

It is always possible, in any reasoning or body of thought, to find presumptions which that reasoning or body of thought cannot itself justify. There are always unfounded presumptions … and we can ‘reflexively,’ make these assumptions the focus of enquiry.^77^

Reflexivity is very much a part of this move to examine identity, to stress situated knowledge, and to bring out its implications for scholarship. Everybody is situated, everybody has a (different) partial view, any pretence of omniscience becomes deeply unfashionable. We are all inside our own frames of reference. Hacking acknowledges this point in *The Taming of Chance* (1990) when he observes that ‘styles of reasoning’ (although he is not talking about his own) are ‘curiously self-confirming’.^78^ The presumptions of a certain body of thought form the foundation, and thus the limit, for both Munchausen syndrome and Hacking.

Debates over retrospective diagnosis in the history of medicine make even clearer the temporal and geographical limits of various concepts. An influential approach within the academic study of medical history considers it anachronistic to apply current diagnoses, or ‘concepts of disease’ to illnesses and conditions in the past. Such an application is known as ‘retrospective diagnosis’. Katherine Foxhall explores whether or not the celebrated medieval abbess Hildegard of Bingen could be said to suffer from migranes, and provides a useful summary of the main points of debate.^79^ Did Rameses II die of tuberculosis? Was the madness of King George III caused by porphyria? Did Nietzche have syphilis?^80^ One of the classic statements against retrospective diagnosis is Caroline Walker Bynum’s *Holy Feast and Holy Fast* (1987) which is sceptical of the utility of describing medieval women as anorexic.^81^ Foxhall changes tack, and argues instead that ‘taking examples of retrospective diagnoses as historical artefacts in themselves’, can be very revealing. This move, putting methodological approaches into historical context, shares much with the approach of the present article.

Adrian Wilson points out that when current concepts are applied across history, and ‘diseases are all taken to coincide with their respective modern concepts’ the effect ‘is to construct a conceptual space in which the historicity of all disease-concepts, whether past or present, has been obliterated’.^82^ Thus diseases are denied any meaningful history, as they are deemed essentially unchanging and always equivalent to the current concept or idea. Hacking is ambivalent about such ‘retroactive’ processes. He argues that ‘[a]s a cautious philosopher, I am inclined to say that many retroactive redescriptions are neither definitely correct or definitely incorrect’.^83^ Whilst such an action might be politically useful, even politically progressive in some senses, it falls some way short of being adequately *historical*. Hacking hedges his bets on this point: ‘[a]s we recede into the past, culture and norms become increasingly different, and I develop qualms about retroactive application’.^84^

Lorraine Daston expands upon Hacking’s sense of history in a review of the second edition of his book *The Emergence of Probability* in 2006. She argues (admittedly talking about his histories of science, not of subjectivity) that his work ‘is not hostile to context … but neither is it about context’. Within this generous review she continues that ‘Hacking is a scrupulous reader, with a strong sense of the otherness of the past, but he does not hesitate to translate seventeenth-century ideas into modern parlance’.^85^ And where does Daston go for an illuminating comparison to drive home the point? Twentieth-century anthropology:

Hacking’s attitude toward his historical actors is endearingly reminiscent of that which Clifford Geertz ascribed to the great British anthropologist E. Evans-Pritchard: however outlandish the beliefs of another people may seem … these strangers ultimately navigate by the same matter-of-fact, rational principles as one’s neighbors in Oxford or Cambridge.^86^

For Hacking, in the assessments of his most generous critics, the past is indeed a foreign country. But it is a particular kind of foreign country, with a sense of difference and sameness imported from a particular anthropological project; it is just as situated and embedded as in any disease category. It is a vision of difference that is founded upon a plastic sameness, an historically specific vision of human nature and of the past.

It is relatively uncontroversial to argue that retrospective diagnosis (as a specifically medical or clinical genre of retroactive redescription) is not simply inadequate history. It also naturalises the present, by projecting currently valid knowledge back through time. If the categories, concepts and ideas that are dominant today can also be found throughout history, then this puts them into a category close to universal, and forecloses any effort to contest or change them. Instead of accepting this damaging stasis, we should follow Joan Scott’s call to give concepts a more situated history, ‘placing them in time and subject to review’, opening the way to change.^87^

The point worth pursuing here is this: if disease concepts should not be projected back into the past, they why should concepts that describe processes of identity formation and change be valid in the past? It seems anachronistic to speak of Ancient Greeks, or Renaissance Lords ‘making themselves up’ in ways that become visible and meaningful in the twentieth century. This is *not* the same as saying that these past identities are irrevocably fixed or permanent. It is simply to say that there are no grounds for arguing that these identities are malleable or negotiable *in the same ways* as envisaged by twentieth-century philosophy, anthropology, sociology and history of medicine. Ultimately, if we baulk at universalising Munchausen syndrome (and I certainly do), we should be very cautious indeed about presuming ‘making up people’ or ‘looping’ to be universal.

It is not fatal to the usefulness of these concepts that they spring from the same context, and are sustained by the same insights as Munchausen syndrome. However, the argument in this article is built upon the fact that Munchausen syndrome is historically specific, and is intelligible as part of an historically specific context. Therefore it cannot be diagnosed throughout history or function in a universal manner. The reason Munchasuen syndrome has been picked to interrogate Hacking is that not only is it specific, but its specificity is identical to that of ‘making up people’ and ‘looping effects’. The insights that make Munchausen syndrome visible—that identity and social roles are malleable and manipulable, rather than fixed and essential—are precisely those that inform and buttress Hacking. Making up people is the conventional side, Munchausen syndrome the pathological side. If we see Munchausen syndrome like this, why should we expect Hacking’s concepts to *stand outside* of this context?

This has wide implications for the history of medicine. It means that we must be more aware of the specificity of our tools as well as the diagnoses we study. As a discipline, the history of medicine is already aware of this in so many areas: few historians today would use Freud’s tools of the death instinct, Oedipus complex or superego to understand the motivations of Henry VIII. We see these tools as bounded, specific and irreducibly historical. So why not Hacking’s?

This injunction, restricting the validity of ‘making up people’ might be seen as constraining, or even destructive to the ability to write history. It might be argued that all language is situated and historically specific, and that (for example) we can only use early modern language to describe early modern history. I think that this is to overstate the case. I am only arguing here that concepts of (self-)identity need to be restricted. This seems a relatively insignificant change when viewed next to the challenges successfully mounted in recent decades to the assumed universality of the human body or human emotions.^88^ History has not been constrained by these challenges to universalism, on the contrary, it has been enriched.

So whilst this proposed restriction of Hacking might be constraining in a specific and narrow sense, it is liberating in another, opening up another order of questions: how is identity imagined in early modern London? With which concepts did medieval hermits parse or cultivate their sense of self, if at all? Is there a subtle violence in assuming all human identity to be sociologically/anthropologically plastic and malleable?

This article too is a product of a specific historical moment. Scholarship in general seems to be careering towards ever-more neurological and epigenetic visions of humanity, with some sociologists cautiously optimistic about the possibility for collaboration with epigeneticists.^89^ I must say that I am not optimistic that this particular playing field is level, or that collaboration would be mutually enriching (and these are doubts with a significant history).^90^ However, in this context, the specificity of twentieth-century anthropology, and its assumption of the universal plasticity of identity, comes more into focus as a point of contrast.

Returning briefly to Derek Freeman’s assessment of Mead and Boas (published in 2000), we can see clear evidence of this. He calls this ‘culturalism’, this idea of malleability in human nature, ‘one of the ruling myths of the twentieth century’. Given the murderous impact of biological thinking and eugenic racism during that 100-year span, one might contest this rather partial characterisation that the twentieth century was ‘ruled’ by a diminishment of the role of biology in human life. In any case, Freeman is now convinced that ‘culturalism’ is buried, because ‘never before have there been such fundamental advances in our understanding of human life’. Predictably, he name-checks the prestigious trio of genetics, cellular biology and neuroscience. Further, he drives home this advantage by reporting the announcement in June 2000 of ‘the virtual completion of the Genome Project’ which allegedly leaves culturalism exposed as ‘one of the most egregious anthropological errors of all time’.^91^ It couldn’t be clearer here that the turn back to biology in the twenty-first century, powered by the rhetorical clout and limitless ambition of the new genetics, genomics and neurosciences, has rendered the culturally-embedded human nature of twentieth-century anthropology more visible. Never mind that the Human Genome project has produced what two scholars have called ‘a list of parts with no instruction book on how to put them together’. In fact the ‘holy grail’ of gene sequencing has thrown up many more questions than answers and few tangible health benefits, let alone insights into the kinds of behaviours studied by anthropologists. When it turned out that humans possessed as many genes as a fruit fly, and shared half their genes with a banana, ‘[t]he molecular biologists who had confidently predicted that all human life could be read off from the linear string of DNA went rather quiet’.^92^ The debate between nature and nurture is not settled—and it is unlikely ever to be—but as the influence of different methodologies wax and wane, the outlines of past systems of thought become less self-evident and more visible.

All concepts have a place in history, but this historical nature becomes visible or obvious in different ways at different times. We should take note of concepts’ place in history, and thus their limits. They might enlighten, they might collapse the past into the present, they might be irrelevant in certain contexts. However, the key to history is awareness that one is telling a story, building a narrative and using a specific conceptual armoury to do so. These narratives and tools have limits. They assume and accentuate, diminish and dismiss various parts of the past as they identify and interrogate source material.

Every history is a history of the present, and thus uses only the tools available at certain points in time. Explicit awareness of, and reflection upon the boundedness of our tools can enrich history with new questions, and caution historians against overreach. In this specific case, we must work through the boundedness and historical specificity of the ‘malleable humanity’, the idea of anthropological, plastic, inscribable human nature. It makes Munchausen syndrome comprehensible as a disordered desire for the ‘sick role’, and underwrites Hacking’s notion of ‘making up people’. A history of this paradoxical ‘anti-universalizing universal’ does not mean that we must join in the swing back to biology. However, we must not give this comfortingly progressive idea of universal plasticity a ‘free pass’ when it comes to historical reflection, lest we commit unwarranted violence on the identity politics of the past as well as the present.

## Footnotes

1 Helen Kinns, D. Housley and D. B. Freeman, ‘Munchausen Syndrome and Factitious Disorder: The Role of the Laboratory in its Detection and Diagnosis’, *Annals of Clinical Biochemistry*, 2013, 50, 194–203, 194.

2 Raman Baweja, Rikita Baweja and Ahamad Hameed, ‘Munchausen’s Syndrome with Rare Hematological Disorder, Systemic Mastocytosis: A Case Report’, *Journal of Neuropsychiatry and Clinical Neuroscience*, 2013, 25, E35–E38, E35.

3 However, the current edition of the International Classification of Diseases (ICD-10) still uses the Munchausen name.

4 Richard Alan John Asher, ‘Munchausen’s Syndrome’, *The Lancet*, 1951, 257, 339–41, 339, 341.

5 Rhodri Hayward, *Transformations of the Psyche in British Primary Care 1880–1970* (London: Bloomsbury, 2014), 35–41; Roger Cooter, ‘Malingering in Modernity: Psychological Scripts and Adversarial Encounters During the First World War’, in Roger Cooter, Mark Harrison and Steve Sturdy, eds, *War, Medicine and Modernity* (Stroud, Gloucestershire: Sutton Publishing, 1999), 125–48; Alan Deacon, *In Search of the Scrounger: The Administration of Unemployment Insurance in Britain, 1920–1931* (London: Bell, 1976).

6 Ludwig Fleck, *Genesis and Development of a Scientific Fact*, trans. F. Bradley and T. J. Trenn, ed. T. J. Trenn and R. K. Merton (Chicago: University of Chicago Press, 1979) [German edition 1935]; Georges Canguilhem, *Le normal et le pathologique* (Paris, 1966) [*The Normal and the Pathological*, trans C. R. Fawcett and R. S. Cohen] (New York: Dordrecht, 1991).

7 Adrian Wilson, ‘On the History of Disease Concepts: The Case of Pleurisy’, *History of Science*, 2000, 38, 271–319.

8 Ian Hacking, ‘Making up People’ in T. Heller *et al*. eds, *Reconstructing Individualism* (Stanford, CA: Stanford University Press, 1986): 222–36; Ian Hacking, ‘The Looping Effects of Human Kinds’, in D. Sperber, D. Premack and A. Premack, eds, *Causal Cognition. An Interdisciplinary Debate* (Oxford: Oxford University Press, 1995), 351–83; Ian Hacking, *Rewriting the Soul: Multiple Personality and the Sciences of Memory* (Princeton, NJ: Princeton University Press, 1995).

9 See for example, Nancy Campbell and Laura Stark, ‘Making up “Vulnerable” People: Human Subjects and the Subjective Experience of Medical Experiment’, *Social History of Medicine*, 2015, 28, 825–48; Ivan Crozier, ‘Making up Koro: Multiplicity, Psychiatry, Culture and Penis-Shrinking Anxieties’, *The History of Medicine and the Allied Sciences*, 2012, 67, 36–70; ‘Made Up People’ conference held at the University of Warwick 25 October 2013 (where Ian Hacking gave the keynote speech, and the argument in this article was first presented): see <http://www2.warwick.ac.uk/fac/arts/history/chm/events/conferences_workshops/madeuppeople/> accessed 20 July 2015.

10 Hacking, ‘Making up People’, 222.

11 Hacking, ‘The Looping Effects of Human Kinds’, 368.

12 Anthony Fry and Tania Gergel, ‘Paternalism and Factitious Disorder: Medical Treatment in Illness Deception’, *Journal of Evaluation in Clinical Practice*, 2016, 22, 565–74, 70. The term Factitious Disorder is popularized by its inclusion in the third edition of the Diagnostic and Statistical Manual of Mental Disorders (DSM-III) (1980). These disorders are defined as ‘characterized by physical or psychological symptoms that are voluntarily initiated by the patient’. S. E. Hyler and Robert L. Spitzer, ‘Hysteria Split Asunder’, *The American Journal of Psychiatry*, 1978, 135, 1502, quoted in Richard A. A. Kanaan and Simon C. Wessely, ‘The Origins of Factitious Disorders’, *History of the Human Sciences*, 2010, 23, 68–85, 70.

13 For an early example of this, see R. Yassa ‘Munchausen’s Syndrome: A Successfully Treated Case’, *Psychosomatics*, 1978, 19, 242–3.

14 Asher, ‘Munchausen’s Syndrome’, 339.

15 David Armstrong, *Political Anatomy of the Body Medical Knowledge in Britain in the Twentieth Century* (Cambridge: Cambridge University Press, 1983), 114.

16 Colin Jones, ‘Raising the Anti: Jan Foudraine, Ronald Laing and Anti-Psychiatry’, in Colin Jones and Roy Porter, eds, *Reassessing Foucault: Power, Medicine and the Body* (London: Routledge, 1998), 286.

17 C. Wright Mills, *White Collar: The American Middle Classes* (New York: Oxford University Press, 1951), xx.

18 David M. Schneider, ‘The Social Dynamics of Physical Disability in Army Basic Training’, *Psychiatry: Interpersonal and Biological Processes*, 1947, 10, 323–33.

19 John Chynoweth Burnham, ‘Why Sociologists Abandoned the Sick Role Concept’, *History of the Human Sciences*, 2013, 27, 70–87, 73.

20 David Armstrong, ‘Actors, Patients and Agency: A Recent History’, *Sociology of Health and Illness*, 2014, 36, 163–74, 163.

21 Erving Goffman, *Asylums: Essays on the Social Situation of Mental Patients and Other Inmates* (New York: Doubleday, 1961); Erving Goffman, *Stigma: Notes on the Management of a Spoiled Identity* (Englewood Cliffs, NJ: Prentice Hall, 1963); Erving Goffman, *The Presentation of Self in Everyday Life* (New York: Anchor, 1959).

22 Michael Pettit, ‘The Con Man as Model Organism: The Methodological Roots of Erving Goffman’s Dramaturgical Self’, *History of the Human Sciences*, 2011, 24, 138–54, 149.

23 Christopher Bass and Peter Halligan, ‘Factitious Disorders and Malingering: Challenges for Clinical Assessment and Management’, *The Lancet*, 2014, 383, 1422–32, 1422.

24 David Mechanic and Edmund H. Volkart, ‘Illness Behavior and Medical Diagnoses’, *Journal of Health and Human Behavior*, 1960, 1, 86–94, 87.

25 See Issy Pilowsky, ‘Abnormal Illness Behavior’, *British Journal of Medical Psychology*, 1969, 42, 347–51. For a brief lineage, tracing sociological influences on hysteria from Parsons, to Mechanic to Pilowsky, see C. D. Marsden, ‘Hysteria—A Neurologist’s View’, *Psychological Medicine*, 1986, 16, 277–88, 285.

26 Martin Roth, ‘The Desire to be Ill’ *University of Durham Medical Gazette*, 1962, 57, 2–14, 10.

27 David J. Vail, ‘Munchausen Returns. A Case Report’, *Psychiatric Quarterly*, 1962, 36, 317–24, 318, 321.

28 A. K. Sehgal and J. C. Mangla, ‘Munchausen Syndrome (a Case Report)’, *Journal of the Association of Physicians of India*, 1964, 12, 309–13, 310.

29 Robert Kemp, ‘The Familiar Face’, *The Lancet*, 1963, 281, 1225.

30 T. L. Dunn, ‘Professional Patients’, *British Medical Journal*, 1964, 5413, 879.

31 Eric Frankel, ‘Munchausen’s Syndrome’, *The Lancet*, 1951, 257, 911.

32 E. Clarke and S. C. Melnick, ‘Munchausen Syndrome or the Problem of Hospital Hoboes’, *American Journal of Medicine*, 1958, 6–12, 6.

33 Henry Werlinder, *Psychopathy: A History of the Concepts: Analysis of the Origin and Development of a Family of Concepts in Psychopathology* (Stockhom: Almqvist & Wiksell, 1978).

34 Maxwell Jones ‘Memorandum of Evidence’, Royal Commission on the Law Relating to Mental Illness and Mental Deficiency, Minutes of Evidence, Thirtieth Day (Tuesday 26 July 1955) (London: HMSO, 1955), 1230 (henceforth Royal Commission, Evidence).

35 British Medical Association, ‘Memorandum of Evidence’, Royal Commission, Evidence, Twenty-Sixth Day (Wednesday 27 April 1955) (London: HMSO, 1955), 1055.

36 Barbara Wootton, *Social Science and Social Pathology* (London: George Allen and Unwin, 1959), 249.

37 Martyn Pickersgill, ‘The Endurance of Uncertainty: Antisociality and Ontological Anarchy in British Psychiatry, 1950–2010’, *Science in Context*, 2014, 27, 143–75, 153.

38 Jones, ‘Memorandum of Evidence’, 1230–1231.

39 British Medical Association, ‘Memorandum of Evidence’, 1054; Royal Medico-Psychological Association ‘Memorandum of Evidence’, Royal Commission, Evidence, Eighth Day (Tuesday 12 October 1954 & Wednesday 27 October 1954) (London: HMSO, 1955), 287.

40 Jones, 'Memorandum of Evidence', 1229, 1236.

41 [Anonymous], ‘Mental Health’, *Medical World*, February 1959, 137–9, 138.

42 Harrison G. Gough, ‘A Sociological Theory of Psychopathy’, *American Journal of Sociology* 1948, 53, 362–3.

43 Pickersgill ‘Endurance of Uncertainty’, 163.

44 For an historical overview of the concept of ‘secondary gain’, see J. J. Van Egmond, ‘The Multiple Meanings of Secondary Gain’, *American Journal of Psychoanalysis*, 2003, 63, 137–47. For ‘imposture’, see Ben Bursten, ‘On Munchausen’s Syndrome’, *Archives of General Psychiatry*, 1965, 13, 264, which draws upon H. Deutsch, ‘The Imposter: Contribution to Ego Psychology of a Type of Psychopath’, *Psychoanalytic Quarterly*, 1955, 24, 483–505. On anexoria nervosa, see J. R. Hawkings, *et al*., ‘Deliberate Disability’, *British Medical Journal*, 1956, 4963, 361–7; Alan Dickson Wright, ‘How Patients have Deceived Me’, *Transactions of the Hunterian Society*, 1955–56, 14, 13–30; Roth, ‘Desire to be Ill’, 1–18. For addiction, see J. C. Barker, ‘The Syndrome of Hospital Addiction (Munchausen Syndrome): A Report on the Investigation of Seven Cases’, *British Journal of Psychiatry*, 1962, 108, 167–82.

45 Ian Hacking, ‘Lost in the Forest’ *London Review of Books*, 2013, 35, 7–8. Online at < http://www.lrb.co.uk/v28/n16/ian-hacking/making-up-people>, accessed 20 July 2015.

46 O. J. Madsen, J. Servan and S. A. Øyen, ‘“I am a philosopher of the particular case”: An Interview with the 2009 Holberg Prizewinner Ian Hacking’, *History of the Human Sciences*, 2006, 26, 32–51, 37.

47 Ian Hacking, ‘Between Michel Foucault and Erving Goffman: Between Discourse in the Abstract and Face-to-face Interaction’, *Economy and Society* 2004, 33, 277–302, 279.

48 Hacking, ‘Looping Effects’, 368.

49
*Ibid.*, 374, 379.

50 Ian Hacking, ‘Biopower and the Avalanche of Printed Numbers’, *Humanities in Society*, 1982, 5, 279–95.

51 Ian Hacking, *The Emergence of Probability: A Philosophical Study of Early Ideas about Probability, Induction and Statistical Inference* (Cambridge, Cambridge University Press, 1975).

52 Michael Lynch, ‘The Contingencies of Social Construction’, *Economy and Society*, 2001, 30, 240–54, 247.

53 Lynch, ‘Contingencies of Social Construction’, 247.

54 Hacking, ‘Between Foucault and Goffman’, 280.

55
*Ibid*., 281.

56 Hacking, ‘Lost in the Forest’.

57 Mararet Mead, *Coming of Age in Samoa: A Study of Adolescence and Sex in Primitive Societies* (Harmondsworth: Penguin, 1943 [1928]), 157–86.

58 Franz Boas in Mead, *Coming of Age in Samoa*, 6.

59 Mead, *Coming of Age in Samoa*, 11.

60 Derek Freeman, ‘Margaret Mead’s *Coming of Age in* Samoa and Boasian Culturalism’, *Politics and the Life Sciences*, 2000, 19, 101–3, 101.

61 Rhodri Hayward, ‘Medicine and the Mind’, in Mark Jackson, ed., *The Oxford Handbook of the History of Medicine* (Oxford, Oxford University Press, 2013), 524–42, 529.

62 For schizophrenia see Jonathan Metzel, *The Protest Psychosis: How Schizophrenia became a Black Disease* (Boston, MA: Beacon, 2009); for syphilis see Fleck, *Genesis and Development of a Scientific Fact*; for pleurisy see Wilson, ‘On the History of Disease Concepts’; for post-traumatic stress disorder, see Young, *Harmony of Illusions*.

63 Asher, ‘Munchausen’s Syndrome’, 339.

64 John Savage, ‘The “Social Life of Methods”: A Critical Introduction’, *Theory, Culture and Society*, 2013, 30, 3–21, 5.

65 Jaques Derrida, *Of Grammatology*, trans Gayatri C. Spivak (Baltimore: Johns Hopkins University Press, 1997 [1967]).

66 Media Education Foundation, ‘Race, the Floating Signifier’ featuring Stuart Hall online at < https://www.mediaed.org/assets/products/407/transcript_407.pdf> accessed 24 July 2015.

67 Anonymous, ‘Compensation for Cupidity’, *The Lancet*, 1961, 277, 1099.

68
*Ibid.*, 1099.

69 Hayward, *Transformation of the Psyche*, 131.

70 Jean Paul Sartre, *Being and Nothingness*, trans Hazel E. Barnes (London: Meuthen & Co., 1957 [1943]), 59.

71 Sartre, *Being and Nothingness*, 60.

72 Hacking, ‘Making up People’, 168.

73 Stephen Greenblatt, *Renaissance Self-Fashioning From Moore to Shakespeare with a New Preface* (Chicago: Chicago University Press, 2005 [1980]), 4.

74
*Ibid.*, 5.

75
*Ibid*., 179.

76 Giles Deleuze and Félix Guattari, *What is Philosophy?*, trans H. Tomlinson and G. Burchill (London: Verso, 1994 [1991]), 16, 27.

77 Roger Smith, ‘The History of Psychological Categories’, *Studies in History and Philosophy of Biological and Biomedical Sciences*, 2005, 36, 55–94, 56.

78 Ian Hacking, *The Taming of Chance* (Cambridge: Cambridge University Press, 1990), 6–8.

79 Katherine Foxhall, ‘Making Modern Migraine Medieval: Men of Science, Hildegard of Bingen and the Life of a Retrospective Diagnosis’, *Medical History*, 2014, 58, 354–74.

80 Roger Cooter, ‘The Life of a Disease?’, *The Lancet*, 2010, 375, 111–12; Sander Gilman, ‘Review of Richard Schain, *The Legend of Nietzsche’s Syphilis’ Isis*’, 2002, 93, 733–4; Bruno Latour, ‘On the Partial Existence of Existing and Nonexisting Objects’, in Lorraine Daston, ed., *Biographies of Scientific Objects* (Chicago: University of Chicago Press, 2000), 247–69.

81 Caroline Walker Bynum, *Holy Feast and Holy Fast: The Significance of Food to Medieval Women* (Berkley: University of California Press, 1987).

82 Wilson, ‘On the History of Disease-concepts’, 273.

83 Hacking, *Rewriting the Soul*, 243. Here Hacking is talking about redescribing soliders shot for ‘cowardice’ in the First World War as suffering from post-traumatic stress disorder (PTSD). Such a redescription—if accepted by the authorities in the late twentieth century—is intended to allow the families of those shot to reclaim medals awarded to those soldiers.

84 Hacking, *Rewriting the Soul*, 254.

85 Lorraine Daston, ‘Review: The History of Emergences’, *Isis*, 2007, 98,801–8, 807.

86 Daston, ‘The History of Emergences’, 807.

87 Joan Wallach Scott, ‘History-writing as Critique’, in K. Jenkins, S. Morgan and A. Munslow, eds, *Manifestos for History* (Abingdon: Routledge, 2007), 19–38, 34–5.

88 Judith Butler, *Bodies that Matter: On the Discursive Limits of Sex* (London, Routledge, 1993); Thomas Dixon, *From Passions to Emotions: the Creation of a Secular Psychological Category* (Cambridge, Cambridge University Press, 2003).

89 Des Fitzgerald, Nikolas Rose and Ilina Singh, ‘Revitalizing Sociology: Urban Life and Mental Illness between History and the Present’, *British Journal of Sociology*, 2016, 67, 138–60.

90 David Armstrong, ‘Tensions in Biopsychosocial Medicine’, *Social Science and Medicine*, 1987, 25, 1213–18.

91 Freeman, ‘Mead’s *Coming of Age*’, 102.

92 Hilary Rose and Steven Rose, *Genes Cells and Brains: The Promethean Promises of the New Biology* (London: Verso, 2012), 278–84.

